# Recent advances and emerging therapies in the non-surgical management of ulcerative colitis

**DOI:** 10.12688/f1000research.15159.1

**Published:** 2018-08-07

**Authors:** Jan Wehkamp, Eduard F. Stange

**Affiliations:** 1 Department of Internal Medicine 1, University of Tübingen, Tübingen, Germany

**Keywords:** ulcerative colitis, therapy, small molecules, biologicals

## Abstract

The so-called “biologicals” (monoclonal antibodies to various inflammatory targets like tumor necrosis factor or integrins) have revolutionized the treatment of inflammatory bowel diseases. In ulcerative colitis, they have an established role in inducing remission in steroid-refractory disease and, thereafter, maintaining remission with or without azathioprine. Nevertheless, their limitations are also obvious: lack of primary response or loss of response during maintenance as well as various, in part severe, side effects. The latter are less frequent in anti-integrin treatment, but efficacy, especially during induction, is delayed. New antibodies as well as small molecules have also demonstrated clinical efficacy and are soon to be licensed for ulcerative colitis. None of these novel drugs seems to be much more effective overall than the competition, but they provide new options in otherwise refractory patients. This increasing complexity requires new algorithms, but it is still premature to outline each drug’s role in future treatment paradigms.

## Introduction

Based on so-called “biologicals” (biological manufacturing process, so far exclusively monoclonal antibodies), the treatment of ulcerative colitis has advanced significantly in recent years. With the introduction of novel small molecules targeting multiple immune effectors, there is more to come. Nevertheless, although all of these new options are nice to have, a cure for this, at times devastating, disease is not on the horizon. Most drug development efforts currently focus on blocking the inflammatory cascade, which most likely is, at least in part, a secondary rather than a primary event. The initial pathogenic level appears to be the slow invasion of commensal bacteria from the colon lumen into the mucosa, facilitated by a mucus barrier dysfunction ^1^.

Much like in Crohn’s disease, the immune response in ulcerative colitis is directed against bacterial antigens, leading to a predominantly mucosal inflammation beginning in the rectum and variably migrating orally to the cecum ^2^. The majority of patients, however, maintain a stable disease extent ^3^. Major clinical symptoms compromising quality of life are diarrhea, abdominal pain, and bloody stools. In the extreme form, a megacolon may develop, although this has become a rare event. Therapy-refractory courses and inflammation-associated colon dysplasia or cancer still require surgery (colectomy), usually with an ileoanal pouch procedure. Extraintestinal manifestations of arthritis, skin disease (pyoderma or erythema nodosum), or ocular inflammation are frequent. The association of colitis with primary sclerosing cholangitis (PSC) is ominous with respect to colon cancer development.

The epidemiology of ulcerative colitis indicates dramatic increases, particularly in industrialized countries like Sweden, where between 1963 and 2005 incidence rose fivefold and prevalence nearly 11-fold to 474/100,000 ^4^. Dramatic rises, for example, in currently industrializing countries of Asia suggest ulcerative colitis is becoming a global disease ^5^. The mechanism(s) behind this rise are obscure and most likely related to environmental factors, since the contribution of genetic predisposition to ulcerative colitis is minor and not subject to such a rapid change.

As a side issue, since most of the novel drugs are quite expensive, the availability of these substances unfortunately will be limited to countries with well-financed health services. In many parts of the world, patients are still treated with standard or locally traditional drugs, if at all.

This review will start with the standards but focus on the now-available monoclonal antibodies directed against tumor necrosis factor (TNF) and integrins as well as the new small molecules likely to be approved soon. Also, fecal microbiota transfer will be dealt with briefly as a drastic alternative. Advances by new treatments are compared to what is already available, and critical evaluation should include a benefit–risk ratio. As a matter of fact, for novel developments, this judgement is limited by the much smaller number of treated patients during a shorter time period and before marketing is even restricted to the published clinical trials. Thus, similar to our previous review on Crohn’s disease ^6^, it is evident that some opinions are just opinions, necessarily subjective, and, admittedly, personal.

## Methods

**Figure 1.**
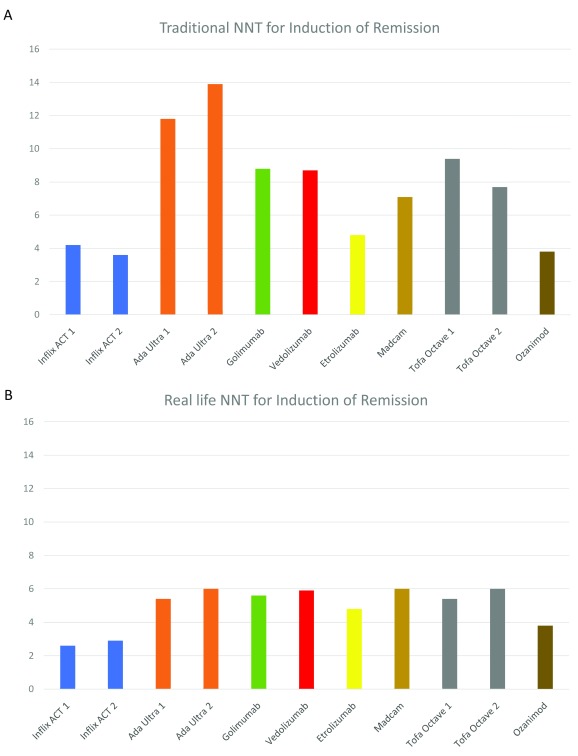
Numbers needed to treat (traditional and real life). **A**. The *traditional* end point of “number needed to treat” ( *t*NNT, number of patients required to obtain one additional remission by the treatment compared to placebo: 100% / difference between treatment and placebo in %). **B**. The *“real life”* NNT ( *rl*NNT, number of patients to achieve one remission including the placebo effect: 100% / total % of patients in remission following treatment). Ada, adalimumab Ultra 1 and Ultra 2 trials; Inflix, infliximab ACT1 and ACT2 trials; Madcam, MAdCAM antibody PF-00547659; Tofa, tofacitinib Octave 1 and 2 trials.

This concise review is non-exhaustive and non-systematic but based on an ongoing PubMed literature screen with the key words “Crohn’s disease” and “ulcerative colitis”. We have focused on the major phase II and III clinical trials to refer to the best evidence available with respect to efficacy. Adverse events are more difficult to judge from this level because of the limited number of patients; here, post-marketing studies are more reliable. To avoid the soft end point of “response” (i.e. patients are better but not well), we prefer clinical remission. It should be noted that in some studies on ulcerative colitis (see below) and most corresponding trials in Crohn’s disease non-responders were excluded in the maintenance phase and just the responders were re-randomized following induction. For the long-term analysis, the responder cohort was reset as 100%, indicating selection bias and skewing data compared to straight-through treatment ^7^. We have also used the *traditional* end point of “number needed to treat” ( *t*NNT, number of patients required to obtain one additional remission by the treatment compared to placebo: 100% / difference between treatment and placebo in %) and compared it to the *real life* NNT ( *rl*NNT, number of patients to achieve one remission including the placebo effect: 100% / total % of patients in remission following treatment). Thus, the *t*NNT refers to the benefit of treatment minus placebo and the *rl*NNT to the overall number of patients required to achieve one remission. In a way, the *t*NNT is of interest to the physician judging the net effect of a drug but the *rl*NNT is important to the patient as a measure of his chances to go into remission. It should be noted that the placebo effect is a well-established and integral part of daily practice and the clinical efficacy from the patient’s perspective is based on a combination of different factors including the isolated drug effect. For most of the drug treatments, both NNTs are given in Figure 1. Please note that comparisons should be made with caution because the studies differ manifold with respect to patient population, timing, end points, and other factors ^7^. Direct head-to-head studies are urgently required but not available.

## Standards

Despite important new developments, the standards still form the basis of our treatment pyramid and the new drugs should be judged against this background of proven effectiveness. These standard therapies including corticosteroids, aminosalicylates, and traditional immunomodulators such as azathioprine and methotrexate as well as calcineurin inhibitors will not be covered in depth. Aminosalicylates and, if necessary, corticosteroids are still the standard treatment during acute relapse. Maintenance usually is covered by aminosalicylates. Steroids are not only ineffective but also associated with significant side effects in long-term treatment.

If aminosalicylates fail, the Cochrane systematic review concluded that azathioprine appears to be more effective than placebo for the maintenance of remission in ulcerative colitis ^8^. Its use in Sweden increased fivefold to 34% from 1976 to 2005 and, in parallel, the colectomy rate decreased ^4^. Concordantly, compared to intolerant patients, those tolerating thiopurines fared much better with respect to colectomy rate, hospital admissions, progression of disease extent, and necessity for anti-TNF therapy ^9^. In England, thiopurine exposure for more than 12 months reduced the likelihood of colectomy by a remarkable 71% ^10^. Although still disputed, some data even indicate protection against dysplasia/colon cancer by aminosalicylates ^11^ and others by thiopurines ^12^. These positive effects are probably due to unspecific anti-inflammatory activity. However, so far, none of these likely benefits of standard long-term therapy have been conclusively shown for anti-TNFs ^13^ or anti-integrins in ulcerative colitis.

The benefit of methotrexate in this disease is disputed, but it may induce steroid-free clinical remission ^14^. On the other hand, ciclosporin is equivalent to infliximab in steroid-refractory acute severe colitis ^15^. The other calcineurin inhibitor, tacrolimus, also appears to be effective ^16^ and may be comparable to anti-TNF agents ^17^, especially in the short term ^18, 19^. Unfortunately, owing to the lack of appropriate business models for older drugs without the possibility of intellectual property protection, calcineurin inhibitors are not licensed for use in ulcerative colitis in Europe.

## Anti-tumor necrosis factor

Two parallel trials (ACT1 and ACT2) proved the efficacy of infliximab, the first anti-TNF antibody, in ulcerative colitis ^20^. The primary end point of “response” (not remission) was achieved in 69% and 64%, respectively, at a dose of 5 mg/kg and in only 37 and 29%, respectively, in placebo patients. Response was defined as a drop but not necessarily normalization of the Mayo score of disease activity. Therefore, this is a much “softer” end point than remission, which was achieved in 39% (ACT1) and 34% (ACT2) compared to 15 and 6% of placebo patients. The resulting *t*NNT for remission was 4.2 or 3.6 in the two studies, respectively, and the *rl*NNT was 2.6 or 2.9. All patients randomized initially in ACT1 and ACT2 were continued after induction and followed on the drug. However, the proportion with sustained clinical remission dropped to 20.5% after 54 weeks despite continued treatment, resulting in a *t*NNT of 7.2 (more than seven patients have to be treated to achieve one additional clinical remission over 54 weeks) and a *rl*NNT of 4.9 (nearly five patients have to be treated to achieve remission up to week 54). At any rate, in ulcerative colitis, there is a majority of non-remitters at 8 weeks, and four out of five patients fail infliximab therapy after 1 year. Also, there is loss of “response” in about 30% of patients between 8 and 54 weeks in ACT1.

The reasons for this loss of response are unclear, but probably both significant intestinal secretion of the drug and immunological neutralization through anti-idiotypic antibody formation are relevant mechanisms ^21– 23^. Particularly with severe inflammation, infliximab appears to be lost from the mucosa, which will likely limit its efficacy. Trough serum levels are a predictive factor of clinical outcome for infliximab treatment in acute ulcerative colitis ^24^ and dose/interval adaptation based on these levels is often helpful. Predictors of early response are younger age and p-ANCA seronegativity ^25^.

On the other hand, since the patients recruited for the two studies were refractory to their concurrent medication, even this limited efficacy is of significant value to the responding patient. Under real life conditions in a Swedish multicenter study, 49% achieved steroid-free clinical remission after 12 months, indicating that proper selection may help improve outcome ^26^. Also, cotreatment with azathioprine may play a role, since it is clinically equivalent to infliximab ^27^, but the combination of both drugs nearly doubles remission rates. Azathioprine but probably not methotrexate may suppress neutralizing antibody formation, and accordingly immunomodulator use is associated with a decreased likelihood of anti-TNF discontinuation ^28^.

The case for adalimumab in ulcerative colitis is much weaker. In two separate studies, drug versus placebo remission rates after 8 weeks were 18.5% versus 10% (Ultra 1) and 16.5% versus 9.3% (Ultra 2), respectively ^29, 30^. This results in a *t*NNT of 11.8 and 13.9, respectively, and the corresponding *rl*NNTs were 5.4 and 6, respectively. The corresponding values for week 52 were 17.3 and 8.5% (placebo), with a *t*NNT of 12.5 and an *rl*NNT of 5.8 ^30^. In infliximab-experienced patients included only in Ultra 2, there was no benefit. Similar to infliximab, trough levels of adalimumab are significantly higher in patients who are in clinical remission and in those with mucosal healing ^31^. However, unlike infliximab, post hoc analysis of six randomized trials in both Crohn’s disease and ulcerative colitis demonstrated no efficacy benefits with immunomodulator plus adalimumab combination therapy ^32^.

Subcutaneous golimumab, the final anti-TNF in this discussion, also may induce and maintain remission in ulcerative colitis. Remission following induction was achieved in 18 versus 6% (placebo), with a *t*NNT of 8.8 at week 6 and an *rl*NNT of 5.6. Maintenance of remission was possible in 23.2–27.8 at the two doses of 50 and 100 mg, respectively, versus 15.6% (placebo) ^33, 34^. Notably, these numbers refer only to the induction responders and not the starting population.

An interesting comparison using network meta-analysis comparing the different anti-TNFs accordingly reveals superior odds ratios for induction of remission with infliximab (5.1) compared to adalimumab (1.8) and golimumab (3.5) in anti-TNF-naïve patients ^35^. In principle, this is a fair comparison because prior futile treatment with another anti-TNF significantly worsens the outcome. In contrast, infliximab was inferior to adalimumab for maintaining remission but was similar to golimumab.

Interestingly, in a cost calculation ^36^, golimumab provided the lowest cost per additional remission ($935) but infliximab was associated with the largest additional number of estimated remissions, although at higher cost ($1,975 per remission). Adalimumab was the most expensive at $7,430 per remission. Since the biosimilars are cheaper and now available, at least for infliximab and soon for adalimumab, costs will drop somewhat, but expenses are still far beyond the standard therapies discussed briefly above.

An unresolved problem is when and how to stop an anti-TNF. Although it should not be a cost problem, discontinuation of an anti-TNF is often requested by patients. The best strategy is unclear, including the continued use of azathioprine or maintenance with an anti-TNF after induction with combination therapy. Taken together in both Crohn’s disease and ulcerative colitis, the relapse rate upon interruption is about 19% per patient year but 80% responded upon re-introduction ^37^. In the end, this will always remain an individual decision personalized to the patient’s prior course.

Side effects of anti-TNFs are manifold and beyond the scope of this discussion. However, it is important to realize that the clinical effect comes at a cost, and in the case of anti-TNFs this largely refers to opportunistic infections, including reactivation of latent tuberculosis, which are sometimes serious and require hospitalization ^38^. An intriguing report from Denmark suggests a much higher risk for serious infections in adalimumab than infliximab users, even fivefold for serious infections requiring hospitalization ^39^. The risk for malignant melanoma is marginally increased by anti-TNF, and the issue of non-Hodgkin lymphoma is controversial. Similarly, it remains unclear whether the statistically increased risk for postoperative complications is real or due to case selection ^40^.

## Anti-integrin

In contrast to anti-TNFs, which affect inflammatory cells by interacting with membrane-linked TNF and inducing apoptosis, the anti-integrins block mucosal entry by interfering with lymphocyte–endothelial cell binding. This leaves already-resident cells in the mucosa intact and may explain the long interval of action: anti-integrins are the azathioprine of the antibodies. Interestingly, recent data actually support the concept that vedolizumab, an anti-integrin with α4β7 antagonism, has substantial effects on innate immunity, including changes in macrophage populations ^41^.

In contrast to natalizumab, which induced cerebral JC-virus infection (progressive multifocal leukoencephalopathy), vedolizumab as an α4β7 antagonist is more intestine specific and appears to be safe in this respect. In the Gemini I trial ^42^ following 6 weeks of induction, remission rates amounted to 16.9 versus 5.4% ( *t*NNT=8.7 and *rl*NNT=5.9). The 47.1% responders were selected to continue and re-randomized, achieving remission after 52 weeks at 41.8% (vedolizumab given every 8 weeks) and 44.8% (given every 4 weeks). If multiplied out, around 21% of the initial starter population might end up in remission, provided that none of the non-responders at week 6 were late remitters. The therapeutic results achieved with vedolizumab following anti-TNF failure were somewhat compromised compared to anti-TNF-naïve patients ^43^. If this is taken into account ^35^, the odds ratio of 4.4 for achieving remission upon induction is in the range of infliximab (5.1) but for maintenance with its odds ratio of 3.6 is superior to all the anti-TNFs (1.2–2.0).

When used “in the real world” as an open medication, one-third of patients with prior anti-TNF failure in France achieved steroid-free clinical remission with vedolizumab ^44^. In Germany, among patients who started vedolizumab for active ulcerative colitis, 25% were in remission at week 54 ^45^. This is quite similar to the remission rates calculated for the starter cohort above. Possibly, the combination of calcineurin inhibitors with vedolizumab may significantly enhance therapeutic action in non or slow responders to the antibody, but experience is limited to small patient series ^46^. Even less is known about vedolizumab combined with anti-TNF ^47^.

The side effect profile of vedolizumab was reassuring in the controlled trials with some cases of nasopharyngitis or arthralgia. Even in the post-marketing studies, it was inconspicuous ^48^, also with respect to enteric infections. A possible signal was a hike in post-surgical complications ^49^, although this was not consistent in other studies. It is probably advisable to establish a drug clearance interval before surgery for anti-TNFs as well as vedolizumab, if clinically feasible.

Abrilumab is another antibody directed against the same target, α4β7, with remission rates in a phase II trial reaching 13.4% compared to 4.4% in the placebo group, but this phase IIb trial is not fully published.

Etrolizumab is also a monoclonal antibody directed against a different target, the β7 subunit of α4β7 and also of αEβ7, which is relevant for epithelial cell cadherin–lymphocyte interactions. Thus, this antibody is less intestine specific but also suppresses intraepithelial lymphocyte retention. Phase III trials in ulcerative colitis are ongoing, but the phase II study was at least promising: no patients in the placebo group had clinical remission at week 10 compared with 21% in the 100 mg group ( *t*NNT=4.8 and *rl*NNT=4.8) and 10% in the 300 mg plus loading dose group ^50^.

Addressing the counterpart of the integrins at the endothelial cell with a MAdCAM antibody, PF-00547659, has also proven effective in a phase II trial ^51^. Lacking a dose response, the maximal remission rate was 16.7% at 22.5 mg compared to 2.7% in the placebo group ( *t*NNT=7.1 and *rl*NNT=6).

Another interesting development is AJM300, an oral inhibitor of α4 which in an induction trial achieved clinical remission in 23.5% compared to 3.9% in the placebo group ^52^. These oral drugs will obviate the immunogenicity that all monoclonal antibodies have in common.

## Janus kinase inhibitors

In contrast to the monoclonal antibodies discussed above, there is also a recent extension of oral small molecule alternatives. The most promising substances to be marketed for ulcerative colitis are the Janus kinase (JAK) inhibitors, the most advanced being tofacitinib. JAKs are intracellular signal mediators activated upon cytokine binding and interacting with the so-called STATs. There are several JAKs, and the currently developed inhibitors differ with respect to their selectivity ^53^.

Tofacitinib inhibits all JAKs but preferentially JAK1 and JAK3. It is already approved in Europe for use in rheumatoid arthritis and, based on the phase III Octave trials ^54^, probably will be licensed for use in ulcerative colitis as well. In the Octave Induction 1 trial, remission at 8 weeks occurred in 18.5% with tofacitinib and in 8.2% in the placebo group ( *t*NNT=9.4 and *rl*NNT=5.4). In the parallel Octave Induction 2 trial, the respective numbers were 16.6 and 3.6% ( *t*NNT=7.7 and *rl*NNT=6). Responders (59.9 and 55% in Octave 1 and 2, respectively) were entered into the “Sustain” maintenance phase, and at 52 weeks remission in this subgroup was achieved in 34–41% depending on dose. Again, if multiplied out with the proportion of responders, about one in four patients starting the trial maintained remission for 1 year. Adverse event signals were observed with respect to non-melanoma skin cancer, cardiovascular events, increased lipid levels, and infections like herpes zoster. More extensive experience with the rheumatological patients suggests that the overall risk of infections and mortality rates appear to be similar to the biological agents and malignancies occurred in the expected range in these rheumatoid arthritis patients ^55, 56^.

Competitors in the field are upadacitinib (JAK1 inhibitor) with a positive phase II trial in ulcerative colitis and filgotinib, another JAK inhibitor, which has been successful in Crohn’s disease, but no data for ulcerative colitis are available.

## Other small molecules

Ozanimod is an oral antagonist of the sphingosine 1 receptor subtypes 1 and 5 that induces peripheral lymphocyte sequestration, potentially preventing them from infiltrating the gastrointestinal tract. In a controlled phase II trial ^57^, ozanimod at week 32 increased the remission rate from 6% in the placebo group to 21–26% ( *t*NNT=5 and *rl*NNT=3.8) in the two doses applied. Blood lymphocyte counts declined by 49% from baseline; the most common side effects were anemia, headache, and bradycardia. The results of the phase III trial are awaited.

A very different approach was the effort to stabilize the colonic mucus level by administration of modified-release phosphatidylcholine (LT-02). After 12 weeks of treatment, remission was achieved in 15% in the placebo group compared with 31.4% in the highest dose group ^58^. This phase II trial was followed by an unpublished negative phase III trial, unfortunately.

There is more to come including phosphodiesterase inhibitors such as apremilast or topically applied oligonucleotides acting as Toll-like receptor 9 agonists, but larger trials are required for qualified judgement.

## Fecal microbiome transfer

A radically different approach, again, is the administration of a heterologous fecal microbiome from healthy donors to ulcerative colitis patients through duodenal/jejunal intubation, colonoscopy, or enemas. A 2,000-year-old hypothesis suggests a modifying role for the microbiome’s composition in disease processes. Overall, most but not all controlled trials and a meta-analysis ^59– 62^ were positive, achieving remission rates significantly above controls, but the remedy is still a black box (maybe brown box is more appropriate). In a remarkable course, a negative trial turned positive when a “super-donor” returned to the study after pausing because of an illness ^59^. The vast majority of those patients receiving his feces (rather than from the other donors) experienced benefit, although the peculiarities of his fecal microbiome are still enigmatic. Possibly, antagonizing the reduced diversity in a patient’s microbiome by increasing bacterial species richness with donor feces may determine clinical success ^63^. However, quite surprisingly, a sterile fecal filtrate was also effective in pseudomembranous colitis, suggesting that the relevant effector is rather the virome including bacteriophages ^64^. Accordingly, there is much to be learned before a “messy” procedure proven to be a cure in many *Clostridium difficile* relapsers becomes standard in ulcerative colitis.

## Outlook: selecting patients, drugs, and end points

It is obvious from this overview that the situation is getting much more complex for both the physician and the patient. Rather than following a straightforward algorithm of (rapid) step up from aminosalicylates to corticosteroids to anti-TNF and azathioprine in order to achieve and maintain (steroid-free) remission, we now have a choice. In a given clinical situation, is anti-TNF appropriate or, because of convincing maintenance and low side effects, is vedolizumab or, in the future, any of the other anti-integrins? Clearly, in severe disease requiring urgent relief, an anti-TNF is more appropriate, but in the longer term loss of response may become a problem. But what about tofacitinib versus azathioprine? Maybe also timing and clinical activity are the key. It is also unclear whether the small molecules now entering the market should generally be used “before” the monoclonals or in combination with them. Nevertheless, with the current lack of comparative trials, all conclusions have to be indirect and speculative.

Moreover, we need a personalized approach because what counts is to have the right choice for the right patient. Unfortunately, there are no reliable tools for the identification of patients at greater risk for a complicated disease course. In addition to the clinical context, molecular approaches to identify likely responders or remitters are currently in focus. At least in Crohn’s disease, the number of membrane-bound TNF-expressing mucosal inflammatory cells is related to the response rate: with higher numbers 92%, and with lower numbers only 15% ^65^. Furthermore, expression of the proinflammatory cytokine oncostatin M is negatively related to the outcome of anti-TNF therapy ^66^. In ulcerative colitis, mucosal α4β7 levels were associated with favorable clinical development during vedolizumab treatment ^67^, and the same holds true for αE gene expression and etrolizumab ^68^. Thus, the selection of patients to improve response rates based on molecular preconditions is at least on the horizon, although certainly not yet as a routine procedure.

Finally, another relevant issue is the antagonistic discussion about end points where regulatory boards prefer patient-related outcome measures and the pharmaceutical industry (and some experts in the field) rather opt for endoscopic or preferably histological remission. On the one hand, there are significant discrepancies between patient-reported outcomes and endoscopic and histological appearances in ulcerative colitis ^69^. There is very limited evidence that treatment should necessarily be escalated until “deep remission” is achieved, although “objective” remission may be prognostically beneficial. The issue is compounded by lacking agreement of the investigators using endoscopic scores ^70^, and the placebo rate, even of endoscopic remission, in ulcerative colitis is a remarkable 23% ^71^. Thus, the use of endoscopy to steer treatment is unsatisfactory. Confocal microscopy may give more solid information but is still not widely available ^72^. Clearly, as a prognostic factor, histology is superior to clinical or endoscopy criteria ^73, 74^, but this does not necessarily imply that histology should guide treatment escalation. Even fecal markers like calprotectin were superior to the Mayo endoscopic score in a study from Japan ^75^. The uncertainties quoted are part of the reason why there is very limited uptake in real-world practice of the “treat to target” recommendations supported by the pharmaceutical industry and some experts ^76^.

In conclusion, the therapeutic options in ulcerative colitis have grown rapidly and will continue to do so. None of the current or currently developed drugs will cure the disease or even attack the initial pathophysiology. Also, there is no drug available or close to marketing which achieves and maintains remission in the majority of patients. Although it is a significant advance to have more alternatives in otherwise treatment-refractory patients, the globally increasing disease of ulcerative colitis remains without real medical remedy, except for surgery (but this is another discussion).
